# Implementation of Cognitive (Neuropsychological) Interventions for Older Adults in Clinical or Community Settings: A Scoping Review

**DOI:** 10.1007/s11065-024-09650-6

**Published:** 2024-11-06

**Authors:** Kerryn E. Pike, Lily Li, Sharon L. Naismith, Alex Bahar-Fuchs, Alessandra Lee, Inga Mehrani, Adam Bentvelzen, Nicola T. Lautenschlager, Megan E. O’Connell, Irene Blackberry, Loren Mowszowski

**Affiliations:** 1 School of Psychology and Public Health, La Trobe University, Melbourne, Australia; 2 John Richards Centre for Rural Ageing Research, La Trobe University, Wodonga, Australia; 3 School of Applied Psychology, Griffith Centre for Mental Health & Menzies Health Institute Queensland, Griffith University, Gold Coast, QLD 4222 Australia; 4 Healthy Brain Ageing Program, Brain and Mind Centre, The University of Sydney, Sydney, Australia; 5 School of Psychology, Faculty of Science, The University of Sydney, Sydney, Australia; 6 School of Psychology, Deakin University, Melbourne, Australia; 7 Department of Psychiatry, University of Melbourne, Melbourne, Australia; 8 Centre for Healthy Brain Ageing (CHeBA), Discipline of Psychiatry and Mental Health, School of Clinical Medicine, University of New South Wales, Sydney, Australia; 9 NorthWestern Mental Health, Melbourne Health, Melbourne, Australia; 10 Department of Psychology & Health Studies, University of Saskatchewan, Saskatchewan, Canada; 11 Care Economy Research Institute, La Trobe University, Wodonga, Australia

**Keywords:** Cognitive stimulation, Cognitive training, Translation, Dementia, Mild cognitive impairment, Subjective cognitive decline

## Abstract

Despite compelling evidence that cognitive interventions for older adults improve cognition, mood, and everyday function, few are implemented in clinical or community practice. This scoping review aims to understand the implementation frameworks and methods used and their contribution to implementation success of cognitive interventions for older adults. We followed the Preferred Reporting Items for Systematic Reviews and Meta-analysis extension for Scoping Reviews (PRISMA-ScR), and searched CINAHL, EMBASE, MEDLINE, and PSYCINFO databases, using terms related to cognitive interventions, implementation, and older adults. This resulted in 5002 studies, of which 29 were included following an iterative process. Most studies reported on implementation of cognitive stimulation for people with dementia. Only four studies used formal implementation frameworks, with three using RE-AIM, and one a process evaluation using complexity theory. The most frequently addressed implementation concepts were Acceptability, Feasibility, and Effectiveness, while Cost, Cost-Effectiveness, and Maintenance were rarely reported. Solutions to common barriers included the importance of good stakeholder relationships and engagement, a manualised intervention flexible enough to adapt to the context, and ensuring facilitators were well-trained, confident, and enthusiastic.

## Introduction

Cognitive decline is a concern for many older adults. It is the cardinal sign of dementia, which affects approximately 55 million people worldwide (World Health Organization, 2021) and causes one of the greatest burdens of illness, injury, and premature death (Australian Institute of Health and Welfare 2024). Mild cognitive impairment (MCI), often considered a prodromal phase to dementia, is also typified by objective cognitive decline, though in the absence of substantial impact on everyday function (Petersen et al., 2014). Older adults with subjective cognitive decline (SCD), but no objective change, are also at increased risk of developing MCI and dementia (Mitchell et al., 2014; Pike et al., 2022).

Across these groups of older adults, substantial research has been conducted into the potential benefits of various cognition-oriented (i.e. neuropsychological) interventions, which aim to address cognitive changes and the resulting impact on daily functioning (see, for example, Bahar-Fuchs et al. (2019); Gavelin et al. (2020); Wong et al. (2023)). Such interventions vary in terms of theoretical approaches, practical techniques, target populations, and outcomes of interest, and with such heterogeneity it is not surprising that terminology in this field is often complex. For the purposes of this review, it is sufficient to describe key features of several common cognition-oriented interventions in ageing. First, the cognitive stimulation approach provides generalised engagement without focusing on any specific cognitive domain or functional skill, and often involves multi-sensory stimulation, reminiscence, reality orientation, and group-based activities (Woods et al., 2023). The structured Cognitive Stimulation Therapy (CST) paradigm (Spector & Orrell, 2006) is a commonly used exemplar of this approach. Cognitive training is another approach which more directly targets functioning in specific cognitive domains via repeated practice of exercises or techniques known to recruit those domains. Cognitive training may involve the use of drill-practice computerised exercises (also known as CCT, see Lampit et al. (2014) for a detailed definition), drill-practice non-digitised exercises (e.g. using paper-and-pencil materials, such as tracing a route on a map or recalling a list of ingredients from a recipe), and/or guided and repeated practice in using compensatory or adaptive strategies, such as systematic use of a diary or using face-name associations for memory functioning. The latter is known as strategy-based training (see Wong et al. (2023) for a detailed description and examples). Yet another commonly used intervention in ageing is cognitive rehabilitation, which offers an individualised, goal-oriented approach aimed at improving daily functioning (see Kudlicka et al. (2023)). Here, a clinician may draw on a variety of compensatory, adaptive, drill-practice, generalised, and/or targeted techniques insofar as they relate to achieving a specific functional task or goal (for illustrative examples, see Clare et al. (2019)).

In addition to improving older adults’ cognition, cognitive interventions are associated with improvements in strategy use, goal attainment, confidence, adjustment, mood, sleep, relationships, engagement in activities, and everyday function (Bahar-Fuchs et al., 2019; Diamond et al., 2015; Gavelin et al., 2020; Kinsella et al., 2016; Kudlicka et al., 2023; Matthews et al., 2020; Pike et al., 2023). Indeed, for older adults with MCI and those without objective impairment, there is sufficient evidence for the World Health Organization (WHO) to recommend the use of cognitive interventions to reduce the risk of cognitive decline and dementia (World Health Organization, 2019). For those with established dementia, several high-quality international Clinical Practice Guidelines recommend the use of cognitive stimulation, supported learning techniques, and compensatory strategies (see Jeon et al. (2023)), with a recent review supporting the benefits of goal-oriented rehabilitation (Kudlicka et al., 2023). Despite this robust evidence base, there remains an enormous evidence-to-practice gap, as these interventions are largely unavailable in clinical practice. In the Australian context, for example, a recent survey of memory clinics revealed that only 20% provided any memory strategy training, despite 74% of respondents identifying cognitive interventions as an important component of adequate post-diagnostic care (Naismith et al., 2022).

Optimal translation of cognitive interventions into routine practice in clinical or community settings (i.e. implementation) can be informed, facilitated, and evaluated by the rapidly developing field of implementation science, which incorporates several different theoretical approaches and frameworks (e.g. Nilsen (2015)). Some implementation frameworks help describe or guide the process of implementation (process models); others aim to explain what influences implementation; yet others evaluate implementation success. Common *process* models include the CIHR (Canadian Institutes of Health Research) Knowledge Translation model (Canadian Institutes of Health Research (CIHR) 2016), the Knowledge-to-Action Framework (Wilson et al., 2011), and the Quality Implementation Framework (Meyers, et al., 2012). Frameworks explaining what *influences* implementation include determinant frameworks, which often look at barriers or enablers impacting implementation outcomes, such as PARIHS (Promoting Action on Research Implementation in Health Services; Kitson et al., 1998), CFIR (Consolidated Framework for Implementation Research; Damschroder et al., 2009), and the Theoretical Domains Framework (Cane et al., 2012). Classic theories (such as the Theory of Planned Behavior; Azjen, 1991) and implementation theories or concepts (such as Organizational Readiness; Weiner, 2009), are also used to understand what influences implementation. Finally, frameworks such as RE-AIM (Reach, Effectiveness, Adoption, Implementation, Maintenance; Glasgow et al., 2019), PRECEDE-PROCEED (Predisposing, Reinforcing and Enabling Constructs in Educational Diagnosis and Evaluation-Policy, Regulatory, and Organizational Constructs in Educational and Environmental Development; Green & Kreuter, 2005), and the framework by Proctor et al. (2011) provide different structures for *evaluating* implementation. The framework by Proctor et al. (2011) proposes eight distinct outcomes for evaluation: acceptability, adoption (or uptake), appropriateness, costs, feasibility, fidelity, penetration, and sustainability.

### The Current Study

To further understand the research-to-practice gap in cognitive interventions for older adults, we aimed to undertake a scoping review of the international literature in this field. The objective of this review was to broadly investigate the methods used in the implementation of cognitive interventions for older adults into clinical practice or community settings, the success or failure of implementation, and how these characteristics and outcomes may differ according to the context (e.g. sample, setting, intervention approach). We considered implementation studies to be studies where an established cognitive intervention approach is being delivered within a clinical or community setting, by people working within that setting (i.e. not someone from the research team). The cognitive interventions usually already have evidence for their efficacy in a research setting (e.g. from a clinical trial). Implementation studies typically focus instead on the process, influences, and success of delivering the intervention within that setting. This was explored through the following primary research questions:

1. Which implementation frameworks (or parts of frameworks) have been used in translating cognitive interventions for older adults?
2. What methods have been used to operationalise these frameworks?
3. What factors have been reported as barriers and enablers of implementation success?

Secondary research questions were:

Do the results differ according to:

- sample (healthy older adult, MCI, dementia, or other)
- setting (inpatient, outpatient health service, community)
- delivery method (in-person, online; individual, group; computerised)
- intervention type (cognitive stimulation, cognitive training, cognitive rehabilitation)
- clinical speciality of person delivering the intervention (neuropsychology, occupational therapy, speech pathology, other allied health, neurology, geriatrics, psychiatry) or layperson (family, volunteer, peer)

## Methods

This review follows the Arksey and O’Malley (2005) approach for scoping reviews, with methodological enhancement by Levac et al. (2010). The stages within this framework are (1) identifying the research question; (2) identifying relevant studies; (3) study selection; (4) charting the data; and (5) collating, summarising, and reporting the results. The review is reported following the Preferred Reporting Items for Systematic Reviews and Meta-analysis extension for Scoping Reviews (PRISMA-ScR) checklist (Tricco et al., 2018).

### Protocol and Registration

The protocol was registered with the Open Science Framework on 12 November 2021 (https://osf.io/yb5ej).

### Eligibility Criteria

Peer-reviewed papers were included if they described both:

- use of a cognitive (i.e. neuropsychological) intervention
- being an implementation study (i.e. translation of cognitive intervention to clinical or community practice)

Exclusion criteria were:

1. non-empirical studies (e.g. review articles, commentaries, letters to the editor)
2. no involvement of older adults (must include some participants > 50 years)
3. published in non-English language
4. full-text unavailable

### Information Sources

To identify potentially relevant documents, the following bibliographic databases were searched from inception to 14th November 2021: CINAHL, EMBASE, MEDLINE, and PSYCINFO. Web of Science was initially also included in the search but returned substantially more papers to screen than the other databases (~ 25,000, compared to between 847 and 1583 for each of the other four databases). Following discussion with a research librarian, we considered that the more general nature of Web of Science meant it was likely to have captured many irrelevant articles, compared to the other four databases focused on allied health, psychology, biomedical, and pharmacology; thus, it was removed from our final search.

### Search

The search strategies were discussed and refined through team discussion, in consultation with a research librarian, who provided ongoing support throughout the search process. The search included combining appropriate terms in each database relating to the following three concepts: cognitive interventions, implementation, and older adults. The final search strategy for each database can be found in Appendix 1. The final search results were exported into EndNote, and duplicates removed.

### Selection of Sources of Evidence

A team of 5 reviewers (LL, KEP, FH, AL, LM) independently conducted title/abstract screening. Reviewers included the senior authors who are both experienced researchers and Clinical Neuropsychologists, and 3 PhD candidates, 2 who are Clinical Neuropsychology registrars. The same 5 reviewers, plus another experienced researcher and Clinical Neuropsychologist (MEO, working in pairs), performed full-text reviews. After the initial title/abstract and full-text reviews, in accordance with the iterative nature of the scoping review process, senior authors (KEP, LM) observed a need to further refine item two of the inclusion criteria (being an implementation study) and re-categorise papers based on their embodiment of “implementation” (see Results section for further detail). Full-text publications were then re-evaluated by KEP and LM according to these refined definitions. Throughout, disagreements on study selection and data extraction were resolved by consensus and discussion with other reviewers.

### Data Charting Process

An online data extraction form within Covidence (Veritas Health Innovation) was jointly developed by three reviewers (LL, KEP, LM). These three reviewers piloted the form on two randomly selected studies by independently extracting the data, discussing results, and continuously updating the data extraction form. Subsequently, data from eligible studies were extracted by a team of 8 reviewers (LL, KEP, AL, LM, MEO, IM, ABF, AB; one reviewer per paper). Any uncertainties during the data extraction process were resolved with discussion and further review of the paper by another author.^1^ 

### Data Items

Data extracted included authors, publication year, sample type, sample size, and country where study was conducted. We also extracted data on various aspects of cognitive interventions including setting, delivery method, intervention type, core aspects, clinician involvement and specialty, outcome effect size estimates, and use of implementation frameworks. Additionally, we gathered information on key implementation components, success and failure measurements, enablers and barriers, stakeholder involvement, outcome conceptualisation, and outcome measures. Health economics data (e.g. cost including resource and equipment use, health-related quality of life, cost-effectiveness) were also extracted where available. The full list of data extraction items can be found in Appendix 2.

### Synthesis of Results

Given this is a scoping review, there was no quantitative statistical model, but rather descriptive data analysis. After extraction, we determined that studies would be best synthesised by type of intervention, and notwithstanding the heterogeneity in terminology within the field, we identified commonalities in approaches and methods to derive the following meaningful groupings:

1. Cognitive stimulation only
2. Cognitive training—drill-practice only
3. Goal-orientated cognitive rehabilitation
4. Cognitive strategy training (often combined with other)

KEP and LM performed a content analysis to extract themes from the included studies regarding barriers and enablers to successful implementation of cognitive interventions in clinical or community settings. A realist framework (Rycroft-Malone et al., 2012) was then used to understand the context and mechanisms that pose as barriers and enablers to the desired outcomes.

## Results

### Selection of Sources of Evidence

**Fig. 1:**
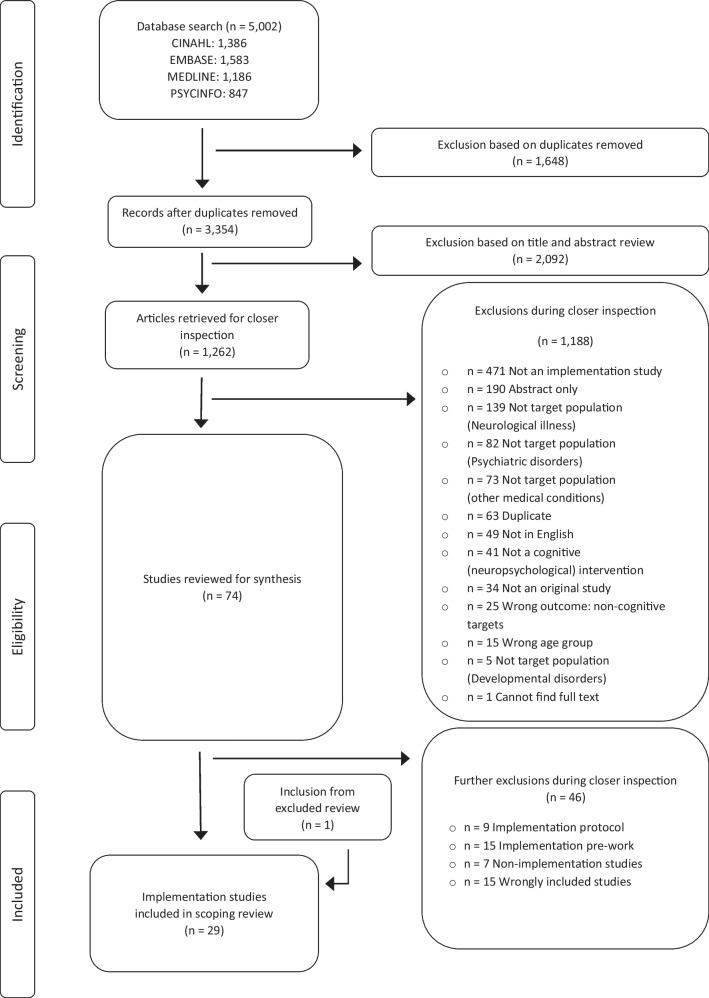
PRISMA diagram of study selection

Following removal of duplicates, a total of 3354 citations were identified from searches of electronic databases and review article references. Based on the title and abstract, 2092 were excluded, leaving 1262 full-text articles to be retrieved and assessed for eligibility. A total of 1188 papers were excluded from the scoping review (see Fig. 1 for breakdown of search results), with the primary reasons for exclusion being not an implementation study (471), not target population (299), or only abstract available (190). Within the 74 remaining studies, as noted above, senior authors KEP and LM identified a need to iteratively refine our core inclusion criterion relating to implementation, to ensure that our review was appropriately focused (per Mak and Thomas (2022) guidelines for conducting a scoping review). We subsequently characterised the remaining 74 studies as outlined in Table 1.

**Table 1:** Re-categorisation of remaining 74 studies

Categorisation	Description	No. of studies
Implementation study	Evaluates the translation of an established intervention to a clinical/community setting, by people working within that setting	28
Implementation pre-work	Describes exploration of concepts or development of interventions, intended to inform down-stream implementation	15
Implementation protocol	Pre-emptive description of an implementation study; work has not yet been carried out	9
Non-implementation studies	Incorporate concepts or terminology overlapping with implementation frameworks (e.g. “feasibility” or “effectiveness”), but the aims and/or methods do not align with focused implementation work	7
Wrongly included studies	Studies which on further examination did not meet inclusion criteria (and should have been excluded earlier on). Examples include a review paper, studies reporting intervention efficacy rather than translation, studies using the word “implementation” in a different context, studies involving implementation of something other than a cognitive intervention (e.g. model of care; clinical education; occupational therapy)	15

One additional study (Mao et al., 2021) was found during this re-evaluation process, from the reference list of an excluded review paper (Cheung & Peri, 2017), taking the total included papers to 29.

### Description of Included Studies

#### Study Population and Setting

Tables 2a and b outline the population and setting for the 29 included studies, divided by intervention type. We split cognitive training into drill-practice exercises only (3 studies) and studies using cognitive strategy training plus other approaches (5 studies). Most included studies (18/29, 62%) used cognitive stimulation (Table 2a), all of which involved persons with dementia. Many of these studies included participants from a residential care setting (8/18; 44%), although implementation within community centres (such as a town hall; 7/18, 39%) or day programs (5/18, 28%) was also common. By comparison, other intervention types (Table 2b) included less cognitively impaired older adults including those with MCI, SCD, or cognitively healthy, in addition to a greater mixture of settings (e.g. community, outpatient, day program, home) not including residential care. For cognitive strategy training, the most common setting was community health (community spaces associated with health, e.g. senior centre 4/5; 80%) with the remaining study in an outpatient health setting. The age groups were predominantly older adults over the age of 65, ranging up to a mean of 83.2 years (Cheung et al., 2019), though some studies involved participants aged over 50 (Beishon et al., 2021) or 60 (Kinsella et al., 2020; Lee, 2016). The most common country represented was the United Kingdom (UK; 10/29 studies; 34.48%), in all categories except cognitive strategy training. Six studies were from the United States of America (6/29; 21%), across all categories except drill-practice exercises, while the eight studies from Asian nations were from all categories except cognitive rehabilitation. There were two studies from Africa (Mkenda et al., 2018; Paddick et al., 2017), and one study each from Australia (Kinsella et al., 2020), New Zealand (Cheung & Peri, 2019), and Portugal (Alvares Pereira et al., 2022). Most studies only used one primary intervention approach; however, those within the cognitive strategy training category all also included psychoeducation. Two of these studies (Lee, 2016; Mao et al., 2021) additionally included cognitive stimulation, drill-practice exercises, and training focused on individual goals, with Mao et al. (2021) also including motor-cognitive exercises.

**Table 2a:** Population and setting characteristics of included cognitive stimulation only studies

First Author (Year)	Sample	Setting	Country	Age Group M (SD or range)	Sample Size (*N*)	*N* Sites
Streater (2016)	Dementia	Residential care	UK	N/R	68	14
Clark (2017)	Dementia; HOA; Stroke; Frailty	Residential care	UK	> 80	N/R	50
Kwak (2021)	Dementia	Residential care	USA	N/R	N/R	161
Tompkins (2020)	Dementia	Residential care	USA	N/R	11	2
Cheung (2019)	Dementia	Community day program	Hong Kong	83.2 (7.2)	20	2
Raghuraman (2017)	Dementia	Community day program	India	76.4 (60-84)	9	1
Tuppen (2012)	Dementia	Community	UK	N/R	12	2
Paddick (2017)	Dementia	Community	Tanzania	80.0 (77–85)	34	6
Mkenda (2018)	Dementia	Community	Nigeria; Tanzania	75 (66–82)^a^; 82 (77–85)^b^	23	2
McAulay (2020)	Dementia; Delirium	Inpatient	UK	N/R	N/R	2
Alvares Pereira (2022)	Dementia	Inpatient	Portugal	> 80.0	6	1
Lundy (2021)	Dementia MCI; SCD; HOA	Outpatient health	USA	> 64	150	1
LaRue (2013)	Dementia	Home	USA	> 65 years	42	N/R
Streater (2017)	Dementia	Mixed^c^	UK	N/R	N/R	63
Orrell (2017)	Dementia	Mixed^d^	UK	80 (48–92)	89	11
Dickinson (2017)	Dementia	Mixed^e^	UK	N/R	N/R	4
Cheung & Peri (2019)	Dementia	Mixed^f^	New Zealand	N/R	214	10
Wong (2018)	Dementia	Mixed^c^	Hong Kong	81.5 (5.9)	30	3

*MCI* mild cognitive impairment; *HOA* healthy older adults; *SCD* subjective cognitive decline; *UK* United Kingdom; *USA* United States of America; *N/R* not reported. ^a^Nigerian sample. ^b^Tanzanian sample. ^c^Residential care, community. ^d^Residential care homes, day centres, NHS trusts. ^e^Day program, outpatient health, community mental health. ^f^Residential care, community health, educational institutes, dementia day programmes

**Table 2b:** Population and setting characteristics of drill-practice exercises, cognitive rehabilitation, and cognitive strategy training studies

First Author (Year)	Sample	Setting	Country	Age Group M (SD or range)	Sample Size (*N*)	Number of Sites	Additional components to primary approach
*Drill-practice exercises only*							
Beishon (2021)	Dementia; MCI; HOA	Home	UK	> 50.0	37	N/R	-
Ng (2021)	HOA; SCD	Mixed^a^	Singapore	75.6 (9.0)	194	23	-
Yeo (2021)	HOA	Community health	Singapore	68.8 (6.3)	94	1	-
*Goal-oriented cognitive rehabilitation*							
Clare (2019)	Dementia	Home	UK	78.6 (7.07)	474	8	-
Morgan-Trimmer (2021)	Dementia	Home	UK	N/R	51	8	-
Lu (2013)	MCI	Mixed^b^	USA	N/R	N/R	1	-
*Cognitive strategy training (often combined with other)*							
Nomura (2009)	Dementia	Community health	Japan	78.9 (6.0)	57	1	Psychoeducation
Mao (2021)	Dementia; MCI	Community health	Taiwan	78.26 (7.0)	130	8	Mixed^c^
Lee (2016)	Dementia; MCI; SCD	Outpatient health	South Korea	> 60	N/R	1	Mixed^d^
Kinsella (2020)	MCI	Community health	Australia	> 60	274	2	Psychoeducation
Felix (2012)	MCI; SCD; HOA	Community health	USA	71.2 (6.6)	112	8	Psychoeducation

*MCI* mild cognitive impairment; *HOA* healthy older adults; *SCD* subjective cognitive decline; *UK* United Kingdom; *USA* United States of America; *N/R* not reported. ^a^Community health, dementia day programs. ^b^Home, educational institute clinic. ^c^Cognitive stimulation, drill-practice exercises, training focused on individual goals, motor-cognitive exercise. ^d^Cognitive stimulation, drill-practice exercises, psychoeducation, training focused on individual goals

#### Intervention Characteristics

Tables 3 and 4 summarise the type and frequency of the cognitive intervention approaches. Across all intervention types, most approaches were conducted in-person (28/29; 97%), with the sole remaining study involving an online brain training game (Beishon et al., 2021). Most interventions were conducted in groups (22/29; 76%). Those interventions that were conducted individually included all three cognitive rehabilitation studies, one of the drill-practice exercise studies (Beishon et al., 2021), and three of the cognitive stimulation studies. One study (Clark et al., 2017) used both an individual and group approach. Only the three studies using drill-practice exercises were computerised (3/29; 10%). Most approaches did not offer monitoring throughout the intervention (23/29; 79%), and it was not offered in any cognitive strategy training approach (see Table 4). Across all intervention types, the session duration ranged from just over 15 min (Clark et al., 2017) to a full day (Cheung & Peri, 2019; Nomura et al., 2009). Similarly, the weekly frequency and overall duration of the intervention approaches described in the included studies ranged considerably.

**Table 3:** Intervention characteristics of cognitive stimulation studies

First author (year)	Intervention name	In-person (Y/N)	Group (Y/N)	Computerised (Y/N)	Monitoring (Y/N)	Session duration (minutes)	Weekly frequency	Overall duration (weeks)
Streater (2016)	CST	Y	Y	N	N	45	2	7
Clark (2017)	Sporting Memories	Y	both	N	N	> 15 min	N/R	N/R
Kwak (2021)	Music and Memory	Y	N	N	N	0–60	1–10	N/R
Tompkins (2020)	Music and Memory	Y	N	N	Y	30–60	1–13	4
Cheung (2019)	Co-S Play	Y	Y	N	Y	45–60	1	8
Raghuraman (2017)	CST	Y	Y	N	N	45	2	7
Tuppen (2012)	Adapted CST	Y	Y	N	N	300	1	Ongoing
Paddick (2017)	CST	Y	Y	N	N	60	2	7
Mkenda (2018)	CST	Y	Y	N	N	60	2	7
McAulay (2020)	CST	Y	Y	N	N	60	1–2^a^	4
Alvares Pereira (2022)	CST	Y	Y	N	Y	45	2	7
Lundy (2021)	CST	Y	Y	N	N	N/A	2	7
LaRue (2013)	LEEPS	Y	N	N	Y	90	2	13–26
Streater (2017)	CST	Y	Y	N	N	45	2	7
Orrell (2017)	CST	Y	Y	N	N	45	2	7
Dickinson (2017)	CST	Y	Y	N	N	45	1	14^b^
Cheung and Peri (2019)	CST	Y	Y	N	N	1 day	1	N/A
Wong (2018)	CST	Y	Y	N	N	45	2	7

*CST* Cognitive Stimulation Therapy; *LEEPS* Elder Rehab Program and Language-Enriched Exercise Plus Socialisation; *N/R* not reported; *N/A* not applicable. ^a^Ward 1: twice per week, Ward 2: once per week. ^b^14 and then 24 for maintenance

**Table 4:** Intervention characteristics of drill-practice exercises, goal-oriented cognitive rehabilitation, and cognitive strategy training approaches

First author (year)	Intervention name	In-person? (Y/N)	Group? (Y/N)	Computerised? (Y/N)	Monitoring? (Y/N)	Session duration (minutes)	Weekly frequency	Overall duration (weeks)
*Drill-practice exercises only*								
Beishon (2021)	Lumosity	N	N	Y	Y	30	5	12
Ng (2021)	ProAge; Neeuro	Y	Y	both	N	60–120^a^	2^b^	24
Yeo (2021)	CCT (NeeuroFIT)	Y	Y	Y	N	120	2	10
*Goal-oriented cognitive rehabilitation*								
Clare (2019)	GREAT	Y	N	N	N	60	10 sessions	12
Morgan- Trimmer (2021)	GREAT	Y	N	N	Y	60	N/A	36
Lu (2013)	DEMA	Y	N	N	N	N/A	Fortnightly	12
*Cognitive strategy training (often combined with other)*								
Nomura (2009)	Cognitive Rehab	Y	Y	N	N	Full day	Monthly	N/R
Mao (2021)	MCI-SET	Y	Y	N	N	120	1	12
Lee (2016)	GCGMS	Y	Y	N	N	N/A	N/A	N/A
Kinsella (2020)	LaTCH	Y	Y	N	N	120	1	6
Felix (2012)	SeniorWISE	Y	Y	N	N	N/R	1^c^	52

*GREAT* Goal-oriented cognitive Rehabilitation in Early-stage Alzheimer’s and related dementias: multicentre single-blind randomised controlled Trial; *N/R* not reported; *N/A* not applicable; *DEMA* Daily Enhancement of Meaningful Activity; *Rehab* rehabilitation; *MCI-SET* Multi-component Cognitive Intervention using Simulated Everyday Tasks; *GCGMS* Goyang Centenarians Good Memory School; *LaTCH* La Trobe – Caulfield Hospital Memory Group Program; *SeniorWISE* SeniorWISE Memory Improvement Program. ^a^90 min (paper and pencil) 60 min for first 12 weeks (CCT) 60 min; 120 min for second 12 weeks (CCT). ^b^Twice per week—first 12 weeks paper and pencil + 60 min CCT; second 12 weeks 2 sessions CCT (60 min, 120 min). ^c^Weekly for 12 weeks then monthly for 9 months

#### Resources Required for Cognitive Interventions

Tables 5 and 6 summarise the resources in terms of materials, facilitators, and staff training needed for each cognitive intervention. All but one of the interventions required the presence of an active facilitator, although facilitators with different backgrounds and qualifications were used. Cognitive stimulation was most often delivered by an occupational therapist (8/18) or direct care worker (8/18), with nurses and psychologists also common facilitators (see Table 5). In preparation for delivering cognitive stimulation, 13 of the 18 studies required their facilitators to attend an in-person (9/13) or online training (4/13) and 6 studies provided a manual as an additional resource. Five studies did not report on training requirements. Furthermore, four studies reported the use of additional resources in the form of an MP3 Player, instruments, arts materials, or sports supplies (e.g. yoga ball).

**Table 5:** Resources required to run the cognitive stimulation interventions

First author (year)	Materials	Facilitator expertise	Preparation for delivering program	Facilitated?
Streater (2016)	Manual	OT	In-person training (1 day); manuals; ongoing support by researchers	Y
Clark (2017)	N/R	DCW	In-person training (1–3 half-days); online discussion forum; ongoing support by researchers	Y
Kwak (2021)	MP3 Player	DCW	N/R	Y
Tompkins (2020)	MP3 Player	DCW	Online training (1 h)	Y
Cheung (2019)	Mixed^a^	DCW	Training provided by coordinator	Y
Raghuraman (2017)	N/R	N/R	N/R	Y
Tuppen (2012)	N/R	Volunteers	N/R	Y
Paddick (2017)	Manual	OT	Training provided by researchers	Y
Mkenda (2018)	N/A	OT; RN; geriatrics	In-person training workshop by researchers (1 day)	Y
McAulay (2020)	Mixed^b^	OT; AC	N/R	Y
Alvares Pereira (2022)	Manual	PSY	Training by following manual	Y
Lundy (2021)	N/R	N/R	In-person training provided by researchers	Y
LaRue (2013)	N/R	Volunteers	Training by following manual and videotapes	Y
Streater (2017)	Manual	PSY; OT	In-person training (1 day), manuals, ongoing support from researchers	Y
Orrell (2017)	Manual	DCW	Training by following manual and DVD, or attendance at training	Y
Dickinson (2017)	N/R	PSY; OT; RN; DCW	N/R	Y
Cheung and Peri (2019)	N/R	Mixed^c^	In-person training workshop by researchers	Y
Wong (2018)	Manual	OT; SW; RN; DCW	In-person training provided by researchers (half-day)	Y

*N/R* not reported; *OT* occupational therapy; *DCW* direct care worker; *RN* registered nurse; *AC* activity coordinator; *PSY* psychology; *SW* social work. ^a^Percussive instrument, rings, yoga ball, bean bags, cards. ^b^Music required for “sounds” session, and art materials required for “being creative” session. ^c^Largest professions were diversional therapists, nurses, occupational therapists, activity coordinators or assistants, healthcare assistants

**Table 6:** Resources required to run the drill-practice exercises, cognitive rehabilitation, and cognitive strategy training interventions

First author (year)	Materials	Facilitator expertise	Training	Facilitated?
*Drill-practice exercises*				
Beishon (2021)	Computer	Self	In-person set-up and demonstration of program at home. Set up guide and troubleshooting manual for FAQs provided	N
Ng (2021)	EEG headband	PSY; Unspec	N/R	Y
Yeo (2021)	Mobile app	Unspec	N/R	Y
*Goal-orientated cognitive rehabilitation*				
Clare (2019)	Manual; Pool Activity Level instrument	OT; RN	In-person training course (2 days), with annual refresher training (1 day) by researchers. Online supervision (monthly, one-on-one), with ad-hoc meetings if needed. Group meetings to share best practice and site consistency	Y
Morgan-Trimmer (2021)	Manual	OT; RN	Training and regular group and individual supervision provided. Practitioner handbook and structured protocol provided	Y
Lu (2013)	Self-management toolkit	RN	In-person training (8 h) and ongoing supervision by researchers	Y
*Cognitive strategy training (often combined with other)*				
Nomura (2009)	Cooking resources, external aids (e.g. labels)	SW; RN; OT; DCW; Unspec	N/R	Y
Mao (2021)	Manual	OT	In-person training (12 h). Online conference attendance (weekly) to discuss participant experience and adjust activities	Y
Lee (2016)	N/R	Unspec	N/R	Y
Kinsella (2020)	N/R	Unspec	Training through introduction to program, program manual, and co-leading group with experienced member. Ongoing supervision provided (weekly). Train the trainer model for further training	Y
Felix (2012)	N/R	LHE	N/R	Y

*Self* self-administered; *PSY* psychology; *Unspec* unspecified; *OT* occupational therapy; *RN* registered nurse; *SW* social work; *DCW* direct care worker; *LHE* lay health educators

Less detail is reported in the three studies using the drill-practice exercises (see Table 6). For the computerised cognitive training in both Ng et al. (2021) and Yeo et al. (2021), they reported using “trained” facilitators but did not provide further detail regarding background expertise, or the training provided to these facilitators. Ng et al. (2021) also had a non-computerised training component, which was delivered by a psychologist, but again did not describe training to deliver the intervention. The remaining study was not facilitated, but a member of the study team attended the participants’ home to familiarise the participant with the intervention set-up. Various materials were required to conduct specific interventions, including a computer, electroencephalogram headband, and mobile app.

The cognitive rehabilitation approaches (see Table 6) all required a clinically trained facilitator, and all utilised registered nurses, with two studies also utilising occupational therapists. All interventions provided manuals, comprehensive in-person facilitator training, and ongoing supervision. Clare et al. (2019) also used the Pool Activity Level instrument (Pool, 2012), a checklist completed with the caregiver to assist with planning and implementing the intervention.

Similarly, all cognitive strategy training studies (see Table 6) required a facilitator, including occupational therapists, nurses, social workers, direct care workers, and one used lay health educators (Felix et al., 2012). Only two of the five studies described facilitator training, with both reporting initial training, followed by ongoing supervision or conferences, and manuals available. One study noted that cooking resources (e.g. ingredients, equipment) were needed.

### What Implementation Frameworks or Parts of Frameworks Have Been Used?

Of the 29 included studies, only four used structured implementation frameworks. Three studies used the RE-AIM framework (Felix et al., 2012; Mao et al., 2021; Ng et al., 2021), while another study used a process evaluation, based on complexity theory, rather than examining implementation success (Morgan-Trimmer et al., 2021). Of the studies using RE-AIM, only Ng et al. (2021) evaluated all components of the framework (i.e. Reach, Effectiveness, Adoption, Implementation (Fidelity, Cost), and Maintenance, as detailed below).

Regardless of whether a structured framework was used, all included studies referred to key implementation concepts or components. We observed inconsistencies, however, in the use of implementation terms, with several studies using different terms to describe the same method or outcome, or studies operationalising terms inconsistently, indicating discrepancies in their intended application of the concepts. This hampered our ability to synthesise and understand patterns across the studies. Aligned with the iterative nature of scoping reviews, we addressed this by creating a detailed description of key implementation concepts, defined in accordance with seminal implementation science resources (Glasgow et al., 2019; Peters et al., 2013; Shepherd et al., 2019) (see Appendix 3). Authors KEP and LM provided the data extraction team with this list, to be used as a common reference point for identifying which implementation components were reported in the included studies.

Tables 7 and 8 describe the components of implementation frameworks reported by broad intervention approach. The most frequently addressed implementation elements were *Acceptability* (90% of studies), *Feasibility* (82%), and *Effectiveness* (76%). Implementation *Barriers* and *Enablers* were each addressed by 55% of included studies. Less frequently addressed elements were *Appropriateness* (48% of studies), *Reach* (45%), *Fidelity* (31%), and *Adoption* (31%). Finally, *Cost* (20%), *Cost-Effectiveness* (10%), and *Maintenance* (6%) were rarely addressed. The patterns of focusing on *Acceptability, Feasibility*, and *Effectiveness* to the exclusion of reporting on *Maintenance*, *Cost*, and *Cost-Effectiveness* were similar across categories of interventions. *Reach* was never reported for studies on cognitive rehabilitation and always reported for studies on drill-practice exercises. *Maintenance* was only assessed in one cognitive stimulation study (Kwak et al., 2021) and one drill-practice exercise study (Ng et al., 2021).

**Table 7:** Implementation components reported for cognitive stimulation studies

First author (year)	Acc	Ado	App	Bar	Cost	C/E	Eff	Enab	Feas	Fid	Main	Reach
Streater (2016)	Y	Y	-	-	-	-	Y	-	Y	-	-	-
Clark (2017)	Y	-	-	Y	-	-	-	Y	Y	-	-	-
Kwak (2021)	Y	-	Y	Y	-	Y	Y	Y	Y	-	Y	-
Tompkins (2020)	Y	Y	-	-	-	-	Y	-	-	-	-	-
Cheung (2019)	Y	-	Y	-	-	-	Y	-	Y	-	-	Y
Raghuraman (2017)	Y	-	Y	Y	-	-	-	Y	Y	Y	-	Y
Tuppen (2012)	Y	Y	Y	Y	Y	-	Y	Y	Y	-	-	-
Paddick (2017)	Y	-	-	Y	Y	Y	Y	Y	Y	-	-	Y
Mkenda (2018)	Y	-	Y	Y	-	-	-	Y	Y	-	-	-
McAulay (2020)	Y	-	-	-	-	-	-	-	-	-	-	-
Alvares Pereira (2022)	Y	-	Y	Y	-	-	-	Y	-	-	-	-
Lundy (2021)	Y	-	-	-	-	-	Y	Y	Y	-	-	-
LaRue (2013)	-	-	-	-	-	-	Y	-	Y	Y	-	Y
Streater (2017)	Y	Y	-	-	-	-	Y	-	Y	-	-	-
Orrell (2017)	Y	-	Y	Y	-	-	Y	Y	Y	Y	-	Y
Dickinson (2017)	Y	Y	Y	Y	-	-	Y	Y	Y	-	-	-
Cheung and Peri (2019)	Y	Y	-	Y	-	-	Y	-	Y	-	-	Y
Wong (2018)	Y	-	Y	Y	-	-	Y	Y	Y	Y	-	Y

*Acc* Acceptability; *Ado* Adoption; *App* Appropriateness; *Bar* Barriers; *C/E* Cost effectiveness; *Eff* Effectiveness; *Enab* Enablers; *Feas* Feasibility; *Fid* Fidelity; *Main* Maintenance

**Table 8:** Implementation components reported for drill-practice exercises, goal-oriented cognitive rehabilitation, and cognitive strategy training studies

First author (year)	Acc	Ado	App	Bar	Cost	C/E	Eff	Enab	Feas	Fid	Main	Reach
*Drill-practice exercises only*												
Beishon (2021)	Y	-	Y	Y	Y	-	Y	Y	Y	-	-	Y
Ng (2021)	Y	Y	-	-	-	-	Y	-	Y	Y	Y	Y
Yeo (2021)	Y	Y	-	Y	-	-	Y	Y	Y	Y	-	Y
*Goal-orientated cognitive rehabilitation*												
Clare (2019)	Y	-	-	Y	Y	Y	Y	Y	Y	Y	-	-
Morgan-Trimmer (2021)	Y	-	Y	-	-	-	Y	-	Y	Y	-	-
Lu (2013)	-	-	-	-	Y	-	-	-	Y	Y	-	-
*Cognitive strategy training (often combined with other)*												
Nomura (2009)	Y	Y	Y	-	-	-	Y	-	Y	-	-	Y
Mao (2021)	Y	-	Y	-	Y	-	Y	-	Y	Y	-	Y
Lee (2016)	Y	-	-	Y	-	-	Y	Y	-	-	-	-
Kinsella (2020)	Y	-	Y	Y	-	-	Y	Y	Y	-	-	-
Felix (2012)	-	-	-	-	-	-	-	-	-	-	-	Y

*Acc* Acceptability; *Ado* Adoption; *App* Appropriateness; *Bar* Barriers; *C/E* Cost effectiveness; *Eff* Effectiveness; *Enab* Enablers; *Feas* Feasibility; *Fid* Fidelity; *Main* Maintenance

### How Were the Implementation Elements Conceptualised?

Tables 9 and 10 show how implementation components were operationalised by each study. *Acceptability* was most often determined from obtaining feedback, but this varied across studies in terms of whether this was obtained from the participant, caregiver, intervention facilitator, or service manager (or a combination). Attendance was also used in some studies to measure *Acceptability*. There were no specific patterns across intervention approaches.

**Table 9:** Implementation conceptualisation for cognitive stimulation only studies

Author (year)	Acc	Ado	App	Cost	C/E	Eff	Feas	Fid	Main	Reach
Streater (2016)	Att	Will (s) Train	-	-	-	Know (f) Ap (f) Conf (f)	Compl	-	-	-
Clark (2017)	FB (f)	-	-	-	-	-	Staff (i)	-	-	-
Kwak (2021)	FB(f) FB(s)	-	FB(f) FB(s)	-	FB(f) FB(s)	Sub (f)	Staff (i) Res (i) Staff (t)	-	LT (set)	-
Tompkins (2020)	Att	Train	-	-	-	Know (f) Conf (f) Behav (p)	-	-	-	-
Cheung (2019)	FB (f) Att	-	FB(f)	-	-	Sub (f) Cog (p)	Staff (i) Res (i) Attr	-	-	Enrol
Raghuraman (2017)	FB (p) FB (c) FB (f)	-	FB (e) FB (f)	-	-	-	Staff (i)	Adapt	-	Backr
Tuppen (2012)	FB(f) FB(c) FP(p)	Will(f)	FB(f) FB(c)	Time (f)	-	Know(f) Sub(f) Sub(c)	Staff (i)	-	-	-
Paddick (2017)	Att	-	-	Res/equip (p) Res/equip (s) Time (p) Time (f)	hQoL hEcon	Cog (p) Mood (p) Burden (c) Mood (c) Sub (c)	Staff (i) Res (i) Attr Compl	-	-	Enrol Backgr
Mkenda (2018)	FB (c) FB (p) FB (f)	-	FB (c) FB (p) FB (f)	-	-	-	Staff (i) Res (i) Attr Compl	-	-	-
McAulay (2020)	FB (p) FB (f) Att	-	-	-	-	-	-	-	-	-
Alvares Pereira (2022)	FB (p) FB (f) FB (c)	-	FB (p) FB (f) FB (c)	-	-	-	-	-	-	-
Lundy (2021)	Att	-	-	-	-	Cog (p) Mood (p)	Compl	-	-	Enrol
LaRue (2013)	-	-	-	-	-	Cog (p) Mood (p) Mood (c) Burden (c)	Attr	Adapt	-	Backgr
Streater (2017)	Att	Train	-	-	-	Know (f) Conf (f) Sub (f)	Compl	-	-	-
Orrell (2017)	Att FB (p) FB (f)	-	FB(f)	-	-	Mood (p) Ap (f) Know (f) Conf (f)	Staff (i)	Notes Check	-	Enrol Backgr
Dickinson (2017)	FB(f)	Will (f)	FB (f)	-	-	Tools(f) Ap(f) Sub(f)	Staff (i) Res (i)	-	-	-
Cheung and Peri (2019)	FB (f)	Train Will (f)	-	-	-	Know (f) Conf (f)	Compl	-	-	Enrol
Wong (2018)	FB (c) FB (f) Att	-	FB (c) FB (f)	-	-	Cog (p) Mood (p)	Attr	Adapt	-	Backgr

*Acc* Acceptability; *Ado* Adoption; *App* Appropriateness; *C/E* Cost effectiveness; *Eff* Effectiveness; *Feas* Feasibility; *Fid* Fidelity; *Main* Maintenance. (p) participant-based measure; (c) caregiver-based measure; (f) facilitator-based measure; (s) service-based measure (e.g. managers); (i) intervention; (t) training; (set) setting. *Att*, attendance; *FB*, feedback; *Will*, willingness; *Train*, training uptake; *Time*, log of time; *Res/equip(s)*, log of resource/ equipment used; *hQoL*, health-related quality of life; *hEcon*, health economics metrics; *Know*, knowledge; *Ap*, approach; *Conf*, confidence; *Sub*, subjective perception; *Behav*, behaviour; *Cog*, cognition; *Mood*, including wellbeing and quality of life; *Compl*, program completed as intended; *Staff*, availability of staff; *Res*, availability of resources; *Attr*, attrition; *Adapt*, local adaptation; *Notes*, facilitator notes; *Check*, program checklists; *LT*, long term; *Enrol*, recruitment records; *Backgr*, background factors

**Table 10:** Implementation conceptualisation for drill-practice exercises, goal-oriented cognitive rehabilitation, and cognitive strategy training studies

Author (year)	Acc	Ado	App	Cost	C/E	Eff	Feas	Fid	Main	Reach
*Drill-practice exercises only*										
Beishon (2021)	FB (p) FB (c) Att	-	FB (p) FB (c)	Time (p) Res/equip (p)	-	Cog (p) Mood (p) Conf (p) Sub (s)	Attr	-	-	Tot (p)
Ng (2021)	FB (p) FB (f) FB (s)	Will (s)	-	-	-	Cog (p) Mood (p) ADL (p)	Attr Res (i) Compl	Check	LT (set) LT (i)	Enrol Tot (p)
Yeo (2021)	Att FB (p) FB (f)	Will (s)	-	-	-	Cog (p)	Staff (i) Res (i)	Check	-	Enrol Tot (s)
*Goal-orientated cognitive rehabilitation*										
Clare (2019)	FB (p) FB (c)	-	-	Time (a) Time (f) Time (c) Res/ equip (s) ServUse	hQoL hEcon	Cog (p) ADL (p) Goals (p)	Compl	Notes Check	-	-
Morgan-Trimmer (2021)	FB (p) FB (c) FB (f)	-	FB (f) FB (p) FB (c)	-	-	Sub (s)	Compl	Notes	-	-
Lu (2013)	-	-	-	Time (a) Time (f) Time (p&c) Res/equip (p)	-	-	Staff (i) Res (i) Prac	Audit	-	-
*Cognitive strategy training (often combined with other)*										
Nomura (2009)	Att FB (f) FB (c)	Refs	FB (c) FB (f)	-	-	Cog (p) Mood (p) Sub (f) Sub (c)	Res (i)	-	-	Backgr
Mao (2021)	Att FB (p) FB (f) FB (s)	-	FB (f)	Time (f) Res/equip (s)	-	Cog (p) ADL (p)	Attr Staff (i) Prac Staff (t)	Check	-	Tot (p) Enrol Backgr
Kinsella (2020)	FB (p) FB (f)	-	Fb (f)	-	-	Sub (p) Sub (f) Goals (p)	Staff (i)	-	-	-
Lee (2016)	FB (p)	-	-	-	-	Cog (p) Mood (p) ADL (p)	-	-	-	-
Felix (2012)	-	-	-	-	-	-	-	-	-	Tot(p) Tot(s) Enrol Backgr

*Acc* Acceptability; *Ado* Adoption; *App* Appropriateness; *C/E* Cost effectiveness; *Eff* Effectiveness; *Feas* Feasibility; *Fid* Fidelity; *Main* Maintenance. (p) participant-based measure; (c) caregiver-based measure; (f) facilitator-based measure; (s) service-based measure (e.g. managers); (a) administrative; (i) intervention; (t) training; (set) setting. *Att*, attendance; *FB*, feedback; *Will*, willingness; *Refs*, referrals; *Time*, log of time; *Res/equip(s)*, log of resource/equipment used; *ServUse*, log of service usage; *hQoL*, health-related quality of life; *hEcon*, health economics metrics; *Cog*, cognition; *Mood*, including wellbeing and quality of life; *Conf*, confidence; *Sub*, subjective perception; *ADL*, activities of daily living; *Compl*, program completed as intended; *Staff*, availability of staff; *Res*, availability of resources; *Attr*, attrition; *Prac*, practicality of training schedule; *Notes*, facilitator notes; *Check*, program checklists; *Audit*, audit data; *LT*, long term; *Tot*, total people; *Enrol*, recruitment records; *Backgr*, background factors

Similarly, across intervention approaches, measurement of *Feasibility* often included measures of attrition and completion rates, as well as availability of staff and resources to deliver the intervention. One strategy training study (Mao et al., 2021) and one cognitive rehabilitation study (Lu et al., 2013) measured *Feasibility* in terms of the uptake of training by staff, whereas a cognitive stimulation study by Kwak et al. (2021) examined staff availability for training.

Studies explored *Effectiveness* in terms of objective and subjective intervention effects on outcomes such as cognition, mood, behaviour, quality of life, and activities of daily living, as well as the effectiveness of implementation strategies, such as impact of facilitator training or organizational support on knowledge and confidence in delivering the intervention. Many studies used standardised, psychometric tools—for example, Paddick et al. (2017) utilised the WHO Quality of Life scale and the Hospital Anxiety and Depression scale to measure intervention effects on participant quality of life and mood, the Addenbrooke Cognitive Examination to measure participant cognitive function, and the Zarit Burden Interview to measure effects on caregiver burden. Other methods included subjective appraisal, where stakeholders were asked to rate or describe their impressions of intervention effects. For example, Tompkins et al. (2020) administered a subjective questionnaire to intervention facilitators comprising questions around level of knowledge, preparedness, skill development, organizational support, and perceived benefits for managing clients’ symptoms. Facilitators were required to rate their subjective agreement with each statement. In another example, Beishon et al. (2021) conducted face-to-face interviews with participants and their caregivers to gather subjective, open-ended feedback on perceived performance improvement over the course of the intervention. Studies differed in their approach to collecting effectiveness data from various stakeholders. Studies utilising drill-practice or goal-oriented rehabilitation interventions tended to focus on participant-reported outcomes, whereas studies employing cognitive stimulation and cognitive strategy training more frequently reported caregiver, facilitator, and sometimes also service manager outcomes in addition to participant outcomes.

*Appropriateness* was assessed with feedback regarding fit, compatibility, and relevance most commonly from facilitators, caregivers, and participants when reported, and appeared similar across categories of intervention. *Reach* was assessed with the number of people enrolled, questionnaires about the background of participants (to explore representativeness of the study sample), and the total number of people or services eligible for the intervention. For cognitive stimulation, *Adoption* was surmised from the willingness of facilitators and services to participate in the intervention, and the uptake of training. For drill-practice exercises, *Adoption* was measured by willingness of services, whereas referrals to the intervention was considered in one strategy training study (Nomura et al., 2009).

*Fidelity* was assessed with checklists, facilitator notes, or audit data. Local adaptations of established interventions were also used as a measure of *Fidelity*, but this occurred exclusively for cognitive stimulation (for example, where the intervention was translated into another language, or where examples or exercises were adapted to suit local culture, traditions, or resources). *Cost* was reported across all intervention approaches based on time invested for all stakeholders, and use of resources and equipment. *Cost-Effectiveness* was calculated based on health-related quality of life, health economics, and feedback from facilitators and service. *Maintenance* was evaluated by assessing whether the intervention continued in the setting long term.

### What Has Been Reported to Support Successful Implementation (Enablers), or Impede Its Success (Barriers)?

As described above, most included studies utilised a cognitive stimulation approach for people living with dementia, with a smaller group of studies utilising other intervention approaches or working with other populations. Nonetheless, there were no apparent differences in the types of barriers or enablers reported across the studies. Interestingly, factors that were identified as barriers in one study were often then identified as enablers in other studies, and vice versa. Additionally, some studies described particular factors as being both barriers and enablers to implementation. For example, Kinsella et al. (2020) reported that intervention facilitators described using a program manual as “both an asset and a challenge” (p.e174), as while the clear and organized structure facilitated program delivery, the staff occasionally also felt this limited their flexibility for discussion and strategy practice within the sessions. This context-based variability across and within studies limited our ability to definitively attribute individual factors as barriers or enablers. Rather, during our thematic content analysis, we characterised potential barriers and enablers identified in the studies in relation to one of four over-arching factors associated with *Stakeholders*, the *Service*, the *Intervention*, or to the intervention’s *Reach*, as shown in Table 11.

**Table 11:**
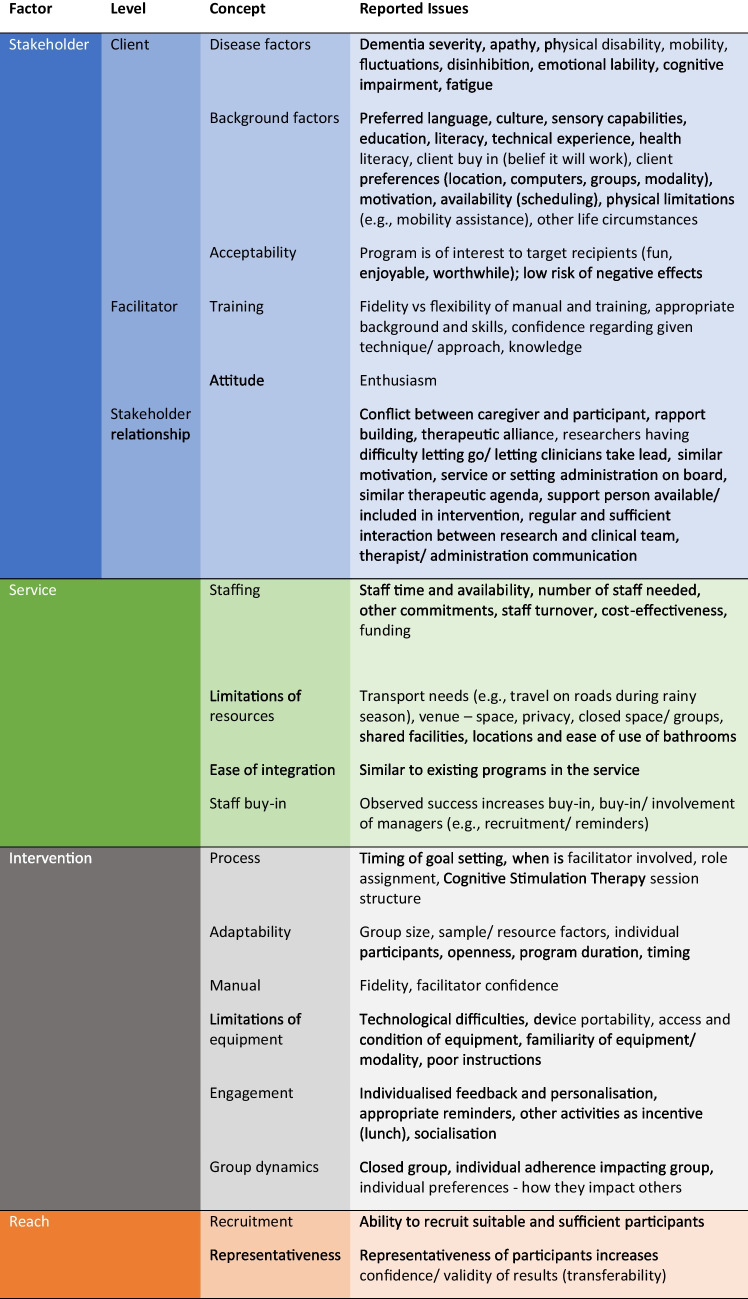
Overview of issues influencing success of cognitive intervention implementation (barriers and/or enablers)

#### Stakeholder Factors

Regarding *Stakeholders*, barriers related to *Client* factors were frequently reported. One of the important client factors was *Background*, including level of education and literacy, technical experience (particularly for computerised tasks), availability, and sensory impairments (e.g. vision or hearing). Many studies commented on cultural factors, for example ensuring that the intervention did not seem “childish” (Raghuraman et al., 2017), or noting that more pragmatic topics such as food or creative production were more acceptable to Chinese participants than more abstract topics (Wong et al., 2018). An often-reported enabler for managing these cultural background factors was the *Adaptability* of the intervention (further considered under *Intervention*), for example the ability to alter the material to account for illiteracy or sensory issues (e.g. Alvares Pereira et al., 2022), or local factors (e.g. Clark et al., 2017). *Acceptability* of the intervention to clients was an important enabler, including that the intervention was enjoyable and had a low chance of negative effects (Tompkins et al., 2020). Another group of client-related barriers were those related to *Disease* factors, for example dementia severity, cognitive impairment, and behavioural symptoms (such as emotional lability, disinhibition, or apathy). They also related to physical disability, particularly impacting mobility.

*Facilitator-related* factors were also frequently reported as barriers or enablers. Facilitator *Training* including appropriate background, skills, and pre-existing knowledge was identified as an important enabler, particularly for understanding common symptoms of dementia and how to manage them, addressing some of the *Disease*-related barriers. Facilitator confidence in a specific technique was noted as an enabler. The issue of balancing fidelity versus flexibility in intervention manuals and other forms of training was noted as both a barrier and an enabler (e.g. Kinsella et al., 2020). Facilitator *Attitude* was important, with enthusiasm for the intervention highlighted as an enabler (Clark et al., 2017). Finally, *Stakeholder Relationships* could be important enablers or barriers and were noted across all levels of stakeholders. For example, the rapport and therapeutic alliance built between clients and facilitators was identified as an important enabler (Clare et al., 2019), whereas conflict between the client and their caregiver (for example, differing levels of engagement in the intervention) was reported as a barrier (Beishon et al., 2021). The relationships between researchers and the clinicians delivering the intervention could enable implementation (Kinsella et al., 2020; Lundy et al., 2021), but could also be a barrier, for example if researchers had difficulties “letting go” of their program and allowing clinicians to work autonomously (Nomura et al., 2009). The importance of getting administrative staff and managers “on board” was also noted (Mao et al., 2021).

#### Service Factors

Staff buy-in was an important enabler reported as part of the *Service* factors. Buy-in from managers was identified as critical, and strategies such as having managers involved with recruitment and sending reminders were successful (Ng et al., 2021). Staff buy-in was reported to be increased by successful experiences of the intervention. *Ease of Integration* of the intervention within the service was another enabler, for example interventions sharing commonalities with existing programs in the service were reported to be more easily implemented (Cheung et al., 2019). *Staffing*—particularly staff availability and turnover, cost-effectiveness, and availability of funding—were often reported as barriers. *Limitations of Resources* were potential barriers, although these could often be managed, including considering the space and privacy of the venue, location and ease of use of bathrooms, and transport needs of clients. The local setting was important, with one study noting the issue of extended travel on roads during the rainy season, which meant difficulties with starting sessions on time, although this was accepted by participants (Paddick et al., 2017).

#### Intervention Factors

In terms of factors related to the *Intervention*, as previously noted, *Adaptability* was an enabler, including flexibility around group size, resources required, program duration, and timing. *Manualised* interventions were reported to increase fidelity and facilitator confidence. The intervention *Process*, regarding role assignment, timing of facilitator involvement and goal setting (Clare et al., 2019), and session structure (Mkenda et al., 2018), could be seen as enablers or barriers. *Group Dynamics* such as whether the group was open or closed, and the impact of individual members’ preferences and adherence impacting on others, were important for group interventions. *Limitations of Equipment* was a clear barrier, particularly technological difficulties, device portability, familiarity with the equipment or modality, and clarity of instructions. *Engagement* was an enabler including the ability to provide individualised feedback and personalisation, to send appropriate reminders (e.g. text messages, Mao et al., 2021), and including time for socialisation and activities such as lunch as an incentive (Mao et al., 2021).

#### Reach

Finally, two factors were noted in terms of *Reach*. *Recruitment* was noted as a barrier, particularly recruiting sufficient suitable people with dementia (Cheung & Peri, 2019). *Representativeness* of trial participants relative to the wider community who would be targeted for the intervention was noted as an enabler, providing increased confidence in the validity of the results and transferability to other settings (Paddick et al., 2017).

#### Understanding Barriers and Enablers: A Realist Approach

**Fig. 2:**
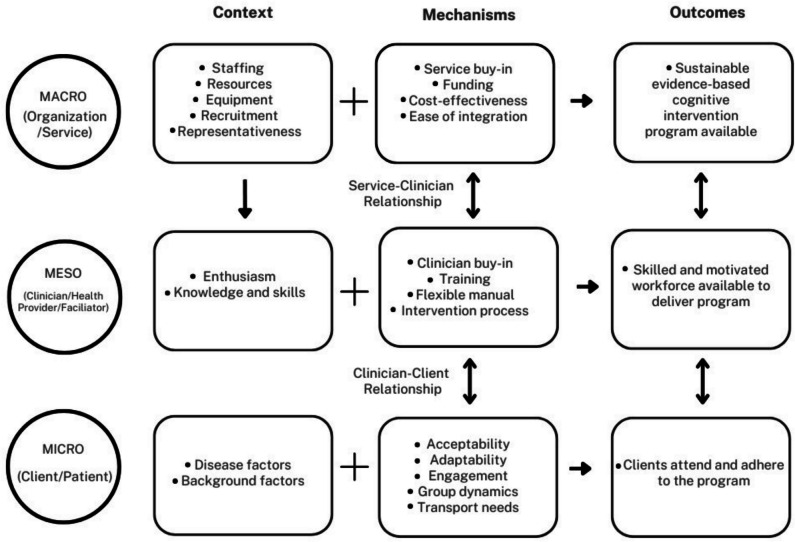
Realist approach to understanding context, mechanisms, and outcomes of reported barriers and enablers

We contemplated how these identified barriers and enablers interact with one another, using a realist approach (Rycroft-Malone et al., 2012) to consider the context, mechanisms, and outcomes at each of the micro (client/patient), meso (clinician/health provider/facilitator), and macro (organizational/service) levels. As shown in Fig. 2, contextual factors and mechanisms at all levels interact with one another to produce the desired outcomes. Starting at the base of our model, at the client (micro) level, disease and background factors are important variables that will impact successful attendance and adherence to the program (outcome), and are impacted by the acceptability of the program, how adaptable it is, client engagement, transport needs, and group dynamics. These mechanisms are also impacted by the client–clinician relationship, as well as the client-caregiver relationship, when relevant. At the clinician (meso) level, the facilitator’s enthusiasm for the intervention and their background knowledge and skills interact with the specific intervention training provided, a flexible manual, clinician belief that the intervention is effective, and the intervention process, leading to a skilled and motivated workforce available to deliver the program. This workforce then impacts on client attendance and adherence as well as the availability of a sustainable cognitive intervention within the setting. Retaining clinicians who have appropriate background skills and who are enthusiastic (rather than burnt out) in turn is influenced by the organizational context (macro level), particularly in terms of adequate levels of staffing, resources, and equipment. A strong relationship between clinicians and their management team and administrative staff supports service-level buy-in to the intervention program. Along with adequate funding, evidence of cost-effectiveness, and ease of integration, these organization-level mechanisms lead to a sustainable, evidence-based cognitive intervention program being made available in a given setting.

## Discussion

### Summary of Findings

Despite strong evidence that cognition-oriented (i.e. neuropsychological) interventions can maintain or improve cognitive functioning in older people, such interventions remain largely inaccessible to the community outside of research settings (Naismith et al., 2022; Wong et al., 2023). To better understand this research-to-practice gap, we scoped the international literature, finding 29 studies reporting implementation of neuropsychological interventions for older adults in clinical or community settings. Most of these studies (62%) reported on implementation of cognitive stimulation for people with dementia, with fewer studies reporting on other cognitive intervention approaches or within MCI, SCD, or healthy older adult populations. Only four studies utilised a formal implementation framework to underpin their implementation evaluation. Regardless of whether a formal framework was employed, it was common for studies to include the implementation concepts of acceptability, feasibility, and effectiveness, but rare for studies to evaluate cost, cost-effectiveness, or maintenance over time. Standardised questionnaires were often used for measuring effectiveness from various stakeholder perspectives, but other implementation concepts were typically measured using bespoke questionnaires, interviews with stakeholders, and data logs of time, resource use, recruitment rates, and attrition. Factors impacting successful implementation related to the stakeholders (client, clinician, and organization levels), service, intervention, or the intervention’s reach. Our realist approach outlined the dynamic interactions between these factors and how consideration of the context and mechanisms at the client, clinician, and organization levels underlies successful implementation of a cognitive intervention program, as indicated by client attendance and adherence, availability of a skilled and motivated workforce, and availability of a sustainable, evidence-based program in the organizational setting.

### Comparison of Differences Across Intervention Approaches, Samples, and Settings

Of the 29 included studies, 62% (or 18/29) utilised cognitive stimulation for older people living with dementia, across a broad range of settings including residential care, day programs, community health, inpatient, outpatient, and home. This may partly reflect the extensive work of Spector and colleagues from University College London, in creating the International Cognitive Stimulation Therapy Centre (https://www.ucl.ac.uk/international-cognitive-stimulation-therapy), which facilitates access to their cognitive stimulation program via a manualised approach, centralised training, and ease of adaptation. Three of the 18 cognitive stimulation studies were carried out in the UK by researchers directly associated with Spector’s group, with a further 10 studies describing adaptions of the manualised program for delivery in the UK or other countries.

Comparatively fewer studies (38%, 11/29) implemented other forms of cognitive intervention (i.e. drill-practice exercises, goal-oriented rehabilitation, cognitive strategy training). Those that did use these approaches also took place across a broad range of settings. These studies addressed a broader range of older adults than the cognitive stimulation studies, including those with objective or subjective cognitive decline (MCI or SCD), and cognitively healthy older adults. This follows patterns in research studies, where more cognitively demanding interventions are seen to be more effective and appropriate in those with milder or minimal cognitive decline, due to greater residual or compensatory cognitive resources and greater insight (Mowszowski et al., 2010; Pike & Kinsella, 2019). Nevertheless, the relative scarcity of *implementation* of these interventions compared to cognitive stimulation may reflect greater barriers. While we did not observe clear discrepancies in the types of barriers or enablers reported across studies using different intervention approaches or in different samples, we were limited by the small number of studies in categories other than cognitive stimulation for dementia. Ultimately, the scarcity of implementation studies in cognitively healthy older adults and people with MCI indicates that recommendations (e.g. Ismail et al. (2020); World Health Organization (2019)) to increase accessibility to cognitive interventions for secondary and tertiary dementia prevention have not yet been widely instigated.

### Commonly Used Implementation Frameworks and Concepts

Regarding our primary research question, we found that less than 15% of included studies utilised a formal, structured, or evidence-based implementation framework. Of these, use of the RE-AIM framework (Glasgow et al., 2019) was the most common and appeared to work well for guiding methodology and operationalising outcomes. Nevertheless, only one study utilised the entire RE-AIM framework. Implementation frameworks are most useful when taken in their entirety, to enable understanding of all the important components for successful implementation. Our exploration of upcoming implementation work, as reported across the protocol and pre-work studies identified through our selection process (Appendix 4), showed the use of a wider variety of implementation frameworks, including the CFIR, Knowledge to Action framework, and iPARIHS. This is important as different frameworks enable focus on different aspects of implementation and may be more appropriate for particular contexts or settings. While it is still a minority of studies using formal frameworks (4/24), this trend demonstrates that the importance of using frameworks appears to be better recognised in new or work-in-progress. Overall, the small number of studies using formal frameworks likely indicates a lack of familiarity for researchers who develop and evaluate interventions, and later aim to implement them in clinical or community practice. Although these researchers are well-versed in research design for efficacy studies, they may lack knowledge or experience in implementation science.

Most of the studies included in the review selectively addressed only a few implementation concepts, most commonly acceptability, feasibility, and effectiveness. These are arguably the more well-known or easily understandable implementation concepts to those familiar with traditional empirical research methods. Less frequently included elements such as appropriateness, fidelity, adoption, and reach may be seen as more “technical”, while rarely included concepts such as cost-effectiveness and costs may be difficult to operationalise without health economics expertise. Maintenance relies on longitudinal monitoring, which is often outside the scope of funding and pragmatic timelines. Yet, these outcomes are just as critical for understanding implementation processes, successes, and failures. Even where an intervention is highly acceptable to stakeholders, produces relevant or meaningful effects, and is feasible to operate, sustainable embedment will fail if the intervention is too costly to run in the longer term, if stakeholders do not adopt the intervention, if it does not fit with the organizations’ core values, or if it is not reaching the intended recipients. Looking at the protocol and pre-work studies, there appears to be some upcoming shift away from the focus on effectiveness, with a greater emphasis on cost, although maintenance was still rarely reported. Without an overarching conceptual framework to provide methodological scaffolding, and investigation of all elements of the framework, conclusions regarding the sources of implementation success or failure are inherently limited.

Another complicating factor in interpreting the findings was the marked inconsistency within and across studies in the use of key implementation terminology and in the application of implementation concepts. For example, Tompkins et al. (2020) discuss low implementation fidelity as a key concern, yet they propose to address this by targeting “buy-in” from the care facility administrators and workforce of intervention facilitators, leaving it unclear if they are referring to fidelity, appropriateness, or adoption. We addressed such ambiguities by creating a glossary of implementation concepts, including definitions and practical examples, synthesised from core implementation science publications (Glasgow et al., 2019; Peters et al., 2013; Shepherd et al., 2019). This glossary (Appendix 3) provided a consistent language from which to synthesise findings across the studies. It represents one of the most practical outputs of this scoping review and may be useful as a guide for future research in this area.

### Factors Influencing Implementation Success

In terms of our third research question regarding barriers and enablers to the implementation process, these were explicitly discussed by just over half of the included studies. We observed that factors identified fell within one of four overarching categories related to *Stakeholders*, the *Service*, the *Intervention*, or to the intervention’s *Reach*. Although many common factors were identified across studies, there were often discrepancies in whether the factors were described as a barrier or as an enabler. We used a realist approach (Rycroft-Malone et al., 2012) to understand how these contextual factors and mechanisms interact at each of the client, clinician, and organizational levels, leading to successful implementation outcomes of client attendance and adherence to the program, a skilled and motivated workforce, and the availability of a sustainable, evidence-based cognitive intervention in the setting. Key factors in successful implementation included (a) stakeholder relationships and involvement at all levels; (b) a manualised intervention that was easy to adapt to the local context (particularly in allowing for culturally relevant tasks, examples, and ideas); (c) sufficient funding for necessary staffing, resources, and equipment; and (d) ensuring intervention facilitators were well-trained, confident, and enthusiastic in working with the client group and in delivering the intervention.

### Strengths and Limitations

Our review had several strengths including pre-registration, a systematic approach following scoping review guidelines, and a comprehensive search strategy, which we then reviewed iteratively to ensure focus on the most relevant studies. We also produced a glossary with definitions and examples of common implementation concepts in this context to aid consistency (Appendix 3), as well as a realist approach model to understand the interaction of factors influencing successful implementation outcomes (Fig. 2).

While our search strategy deliberately included broad search terms to capture as much relevant literature as possible, we recognise that our review may be limited by inadvertently missing some pertinent studies. This may in part reflect heterogeneity in implementation terminology within and across fields (e.g. overlapping use of “effectiveness” to describe both treatment efficacy as well as effectiveness of implementation strategies within the target context). Furthermore, due to the lack of knowledge of researchers in this field regarding implementation science and terminology, particularly from more than a few years ago, it may be that studies did not use the type of terminology that would have been identified in our original search. We scrutinised study aims and methodologies during the screening process to differentiate those studies truly focusing on implementation but recognise the possibility of missing some research due to such overlaps. We have attempted to pragmatically address this issue by creating a useful glossary of terms (Appendix 3) that may be taken up by other researchers, particularly those more familiar with traditional experimental research who then become interested in research translation.

Our findings may also be limited by the scarcity of included studies examining populations other than dementia, and those looking at drill-practice exercises, goal-oriented rehabilitation, and cognitive strategy training relative to cognitive stimulation. There were also few studies examining computerised approaches. Although international guidelines (Ismail et al., 2020; World Health Organization, 2019) suggest cognitive interventions should be offered to older adults with MCI, SCD, or no concerns with their cognition, only seven studies included participants with MCI (only two of these were solely MCI); just four studies included participants with SCD (none SCD alone); and six studies included healthy older adults (only one was solely healthy older adults). This means that our conclusions about implementation challenges and ways to surmount these are tentative for these cohorts. Similarly, our findings are tentative for approaches other than cognitive stimulation as only three studies looked at drill-practice approaches, three looked at goal-oriented cognitive rehabilitation, and five explored cognitive strategy training (combined with other approaches).

Another limitation of our review relates to identification of tools to conceptualise and operationalise different implementation concepts. We were hoping to collate a list of standardised or commonly used measures to inform future implementation trials in this field. Unfortunately, however, the only standardised approaches reported in the included studies were standardised tools (predominantly questionnaires) for measuring effectiveness from relevant stakeholders. For most other implementation concepts, data arose from examining recruitment rates, attrition logs, recording time and resource use, bespoke questionnaires, or qualitative interviews with stakeholders. Although not quite as easy as standardised tools to integrate into future implementation studies, the collation of these examples at least provides a basis for creating relevant methods for measuring outcomes.

### Clinical Implications and Future Directions

Buy-in from all stakeholders, and the relationships between stakeholders were important components to implementation success. This suggests that work is needed to champion the effectiveness and need for cognitive interventions at all levels—including the clients, facilitators, and service settings (e.g. memory clinics or residential care settings). This process begins with establishing ongoing relationships between researchers and people working within appropriate organizations. Work into community-based participatory research (e.g. Belone et al. (2016)) demonstrates the complexity of relationships between academia and community, and that these take time and investment from both parties. Successful implementation occurs only after trusted relationships are created, the capacity of the organization—particularly resources, readiness, and priorities—is considered, and there is flexibility and mutual learning from one another (Belone et al., 2016). Often a “bridge person” representing both academia and the community provides a key link (Belone et al., 2016). Effective academic-community partnerships can lead to buy-in at the client, clinician, and organizational level, creating “change champions” who can influence other levels through their relationships with those stakeholders. It may be helpful to target training programs for future clinicians likely to work with older adults in roles where they could potentially deliver these interventions. Currently, many services working with older adults are purely assessment-focused, for example focusing on neuropsychological assessment to determine if the older adult has a diagnosis of dementia. We need to shift the paradigm so that services move beyond assessment to incorporate post-diagnostic care (Jeon et al., 2023), and concurrently to advocate for the critical role that cognitive interventions can play in this space. Advocacy, particularly from stakeholders at the organizational level, is the best hope for policy change that could result in increased funding to provide staffing and other resources to deliver these interventions.

Few studies in this area have used formal implementation frameworks to date, and only one utilised the entire framework, which creates difficulty in evaluating all aspects of a successful implementation. Lack of formal frameworks also creates issues in the consistency of terminology. Going forward, implementation studies should aim to use formal frameworks, and clearly define the concepts and outcomes being used. We are currently applying the learnings from this scoping review to a pilot study which will train clinical neuropsychologists to implement cognitive interventions for people with MCI in memory clinics around Australia.

We also noted that several studies reported local or contextual adaptations of a manualised intervention as an important component of their implementation process (e.g. Paddick et al. (2017) describe accounting for cultural and education differences as well as resource availability in Tanzania). While this adaptation can present as a barrier as it requires time, creation or modification of content, and in-depth knowledge of local needs and preferences, it also presents as an enabler by enhancing the potential for acceptability, adoption, and appropriateness. Ultimately, this may require scoping of local needs and preferences and training in cultural competence for staff involved in planning and implementing interventions, to maximise reach, engagement, and adherence.

## Conclusions

Despite compelling evidence for the benefit of cognitive interventions for older adults, the translation of these programs into clinical practice and community settings has been slow. This is particularly the case for older adults without dementia and for drill-practice, cognitive rehabilitation, and cognitive strategy training intervention approaches. Few studies have used formal implementation frameworks, which can lead to inconsistency in terminology and missed evaluation of important aspects of implementation processes and outcomes. Creating strong stakeholder involvement and relationships across all levels, using manualised interventions that are flexible for adaptation to the local context, and ensuring facilitators receive appropriate training in the client group and intervention so that they are confident and enthusiastic are common enablers of implementation success. Multiple contextual and mechanistic factors at each of the client, clinician, and service levels interact dynamically to aid or hinder implementation success.

## Data Availability

The data used in this scoping review is all presented within the tables.

## Appendix 1

### Final Search Terms

Search strategy across CINAHL, MEDLINE, PSYCINFO, and EMBASE—noted where differences occurred within each database| Search conducted on 14 Nov 2021.

**Table:**

No	Search term
1	cognitive intervention*
2	cognitive training
3	memory training
4	memory intervention*
5	neuropsycholog* intervention*
6	brain training
7	cognitive remediation
8	cognitive rehab*
9	cognitive stimulation
10	/cognitive remediation (for CINAHL, MEDLINE, & PSYCINFO) /cognitive remediation therapy (for EMBASE)
11	/rehabilitation, cognitive (for CINAHL) /cognitive rehabilitation (for EMBASE & PSYCINFO) (not available for MEDLINE)
12	1 or 2 or 3 or 4 or 5 or 6 or 7 or 8 or 9 or 10 or 11 (remove “or 11” for MEDLINE)
13	implement*
14	accept*
15	adopt*
16	feasib*
17	usage
18	usability
19	integrat*
20	deploy*
21	utiliti?*
22	framework*
23	knowledge transfer
24	translat*
25	embed*
26	/implementation science (not available for PSYCINFO)
27	13 or 14 or 15 or 16 or 17 or 18 or 19 or 20 or 21 or 22 or 23 or 24 or 25 or 26 (for CINAHL, MEDLINE, & EMBASE) 13 or 14 or 15 or 16 or 17 or 18 or 19 or 20 or 21 or 22 or 23 or 24 or 25 (for PSYCINFO)
28	12 and 27
29	old* adult*
30	old* age*
31	aging
32	/aging
33	ageing
34	aged
35	/aged (not available for PSYCINFO)
36	dementia
37	/dementia
38	Alzheimer*
39	mild cognitive impairment
40	MCI
41	29 or 30 or 31 or 32 or 33 or 34 or 35 or 36 or 37 or 38 or 39 or 40 (for CINAHL, MEDLINE, & EMBASE) 29 or 30 or 31 or 32 or 33 or 34 or 36 or 37 or 38 or 39 or 40 (for PSYCINFO)
42	28 and 41

## Appendix 2

### List of Extracted Data Items

- author(s)
- year of publication
- origin/country of origin (where the source was published or conducted)
- sample (healthy older adult, mild cognitive impairment, dementia, other) and sample size within the source of evidence
- setting (inpatient, outpatient health service, community health, community ageing/seniors services, other)
- delivery method (in-person, remote (e.g. online); individual, group; computerised)
- intervention type (e.g. cognitive stimulation, cognitive training, cognitive rehabilitation)
- core aspects of the intervention (e.g. duration, frequency, materials, adjunctive components)
- clinical specialty of delivery context (neuropsychology, OT, speech pathology, other allied health, neurology/geriatrics)
- intervention outcome effect size estimates
- whether an implementation framework was identified and used (and which one)
- if an implementation framework was not used—are any key components/concepts/themes identified in common implementation frameworks being used (e.g. feasibility, acceptability, sustainability)
- how was implementation success and failure measured
- was implementation successful
- any reported enablers to implementation success
- any reported barriers to implementation success
- who were the stakeholders
- how were outcomes conceptualised
- what outcomes measures were used
- were findings reported or communicated within the setting
- any reported health economics (costings)

## Appendix 3

### Glossary of Implementation Terms

**Table:**

Component		Examples from our included papers	Short label/code
Effectiveness	The impact of an intervention on important outcomes, including potential negative effects, heterogeneity of effects, and reasons for success or lack of success (Glasgow et al., 2019 – RE-AIM)	Participant changes in cognitive measures/mood (wellbeing, quality of life)/ADLs/goals/confidence/behaviour	Cog (p) Mood (p) ADL (p) Goals (p) Conf (p) Behav (p) Sub (p)
		Facilitator changes in dementia knowledge/approach/tools/sense of competence;	Know (f) Ap (f) Tools (f) Conf (f) Sub (f)
		Caregiver burden/depression;	Burden (c) Mood (c) Sub (c)
		Subjective impressions (sub) of whether the intervention was helpful (participant, caregiver, facilitator, service)—can be obtained via interviews, surveys, etc	Sub (s)
Acceptability	Perceptions among stakeholders (e.g. consumers, providers, managers, policy makers) of intervention being agreeable, palatable, or satisfactory (Shepherd et al., 2019 – Proctor model; Peters et al., 2013a, b)	Feedback—interest, enjoyment (participant, caregiver, facilitator, service). Can be obtained via interview, survey, questionnaires, etc	FB (p) FB (c) FB (f) FB (s)
		Attendance	Att
Appropriateness	Fit, relevance, or compatibility of the intervention for the given setting, provider, or consumer; or for a particular issue or problem (Shepherd et al., 2019 – Proctor model; Peters et al., 2013a, b)	Feedback (participant, caregiver, facilitator, service, experts) focused on fit, compatibility, and relevance. Can be obtained via interview, survey, questionnaires, etc	FB (p) FB (c) FB (f) FB (s) FB (e)
Feasibility	The extent to which an intervention can be successfully carried out in a particular setting or organization (Peters et al., 2013 a, b; Shepherd et al., 2019, Proctor model)	Availability of staff as required to run the intervention (time OR competency)	Staff (i)
		Availability of resources as required to run the intervention	Res (i)
		Practicality of the training program/schedule	Train
		Availability of staff, resources, and trainers to undergo training	Staff (t) Res (t)
		Attrition/retention	Attr
		Program completion as intended within the setting	Compl
Cost	Financial and economic costs of an implementation effort, comprising cost of the intervention components, cost of the implementation strategies used to implement, and costs of delivery within each setting (Proctor et al., 2011)	Log of time devoted to intervention (admin, facilitators, participants, caregivers)	Time (a) Time (f) Time (p) Time (c)
		Log of resource/equipment used for intervention (site, participant)	Res/equip (s) Res/equip (p)
		Log of service usage during intervention period	ServUse
Cost effectiveness	Weighing up of Cost outcomes vs Effectiveness and Feasibility outcomes, to make some comment on value from a cost/benefit perspective	Health-related QoL	hQoL
		Health economics metrics, e.g. quality-adjusted life years; saving/expenditure calculations; projected costs; cost per unit benefit (e.g. MMSE point)	hEcon
Reach	The absolute number, proportion, and representativeness of individuals willing to participate in a given initiative, intervention, or program (Glasgow et al., 2019, RE-AIM), and the extent to which those eligible to benefit from an intervention actually receive it (Peters et al., 2013)	Population/area records—total people with the named condition	Tot (p)
		Site records—total clients with the named condition	Tot(s)
		Recruitment records (number/proportion enrolled)	Enrol
		Questionnaires/interviews documenting background factors (e.g. education, age, socioeconomic, cultural background)	Backgr
Adoption	The number/proportion of settings or interventionists with the intention, initial decision, or action to try/initiate/employ an innovation or evidence-based practice (Proctor et al., 2011) and the representativeness of those willing (Glasgow et al., 2019 RE-AIM)	Information obtained from the site regarding willingness (will); staff facilitators, and non-staff facilitators (e.g. caregivers, volunteers). Can be obtained from motivation-based questionnaires (e.g. ORIC) or interviews	Will(s) Will(f)
		Interventionist training uptake	Train
		Referrals made	Refs
		Qualifying factors describing those who agree vs those who don’t	Facts
Maintenance	The extent to which (a) behaviour is sustained 6 months or more post- intervention for individuals; and (b) a program or policy becomes part of routine organizational practices and policies within the setting after research funding ceases. (Glasgow et al., 2019, RE-AIM)	Number/proportion of settings still delivering the intervention (LT = long term)	LT(s)
		Number/proportion of individuals still using/following the intervention (LT = long term)	LT (i)
Fidelity	The extent to which the intervention is implemented as originally prescribed or intended by the developers (Proctor et al., 2011), and implemented consistently across different settings, staff, and patients (Glasgow et al., 2019 RE-AIM)	Facilitator notes	Notes
		Audit data	Audit
		Protocol/program checklists	Check
		Local adaptations indicating differences from original version	Adapt

*Key for codes*

Generally, the short codes appear next to the example that they represent.

We have used (p) to indicate a participant-based measure (i.e. older adult).

We have used (c) to indicate a caregiver-based measure.

We have used (f) to indicate a facilitator-based measure (referring to intervention facilitators—could be staff, laypeople, etc.).

We have used (s) to indicate a service-based measure (e.g.managers).

We have used (a) to indicate administrative.

For feasibility, (i) refers to intervention and (t) to training.

For maintenance, (i) refers to intervention and (s) to setting.

## Appendix 4

### A Brief Exploration of Trends From Implementation Protocols, Pre-work Studies, or those Mentioning Implementation Concepts

Similar to the “true implementation studies” previously described, the nine implementation protocol studies were mostly focused on dementia (7/9), with more than half describing implementation of cognitive stimulation (5/9). Only two of the protocols reported use of an implementation framework, the Consolidated Framework for Implementation (CFIR; Spector et al., 2019) and the Knowledge to Action framework, along with elements from CFIR (Cooper et al., 2020). *Acceptability* (6/9) and *Feasibility* (7/9) remained commonly included implementation elements, along with *Barriers* (9/9) and *Enablers (7/9)* to implementation*. Effectiveness* was less commonly reported (3/9) than in the studies previously described. *Maintenance* was uncommonly reported (2/9), but *Costs* of the implementation were reported by more than half of the protocols (5/9).

Of the 15 pre-work studies, most (9) focused on dementia. Of note, only 6/15 studies described planned implementation of cognitive stimulation, with an equal number describing drill-practice approaches, and the remaining three describing cognitive strategy training (in conjunction with training focused on individual goals for two of those studies). Two of the pre-work studies used implementation frameworks, the CFIR (Stoner et al., 2020) and the Integrated Promoting Action on Research Implementation in Health Services framework (iPARIHS; Douglas & Afoo, 2019). *Acceptability* (11/15), *Barriers* (11/15), and *Enablers* (10/15) to Implementation were the most reported elements*. Feasibility* was only reported in seven studies, and *Effectiveness* in only three. *Maintenance* was only reported in one study, and *Costs* of the implementation in three.

Finally, the studies that mentioned implementation concepts, but were not true implementation-focused studies, were also primarily in people with dementia (5/7) and focused on cognitive stimulation (5/7). None of these studies used an implementation framework. *Acceptability* and *Feasibility* were the most frequently included implementation concepts, each reported in 4 studies*. Effectiveness* and *Enablers* were reported in 3 studies each, but *Barriers* to implementation were only reported by 2 studies. *Maintenance* (2/7) and *Costs* of the implementation (1/7) were rarely reported.

## Footnotes

1 Covidence (version 2.0)
